# Numerical investigation of the effect of dust shields on accumulation of dust over PV panels

**DOI:** 10.1007/s11356-023-26502-7

**Published:** 2023-03-23

**Authors:** Ramy Shenouda, Mohamed S. Abd-Elhady, Hamdy A. Kandil, Mahmoud M. Dagher

**Affiliations:** 1 Department of Mechatronics, Faculty of Engineering and Materials Science, German University in Cairo (GUC), Cairo, Egypt; 2 Department of Mechanical Engineering, Faculty of Engineering, Beni-Suef University, Beni-Suef, Egypt

**Keywords:** Photovoltaic, Dust mitigation, Dust shields, Air gaps, Modeling dust deposition, CFD

## Abstract

Dust accumulation on photovoltaic panels represents a major challenge for the operation of solar panels especially in the regions known by their high rate of dust and low frequency of rain. The objective of this study is to minimize dust accumulation on PV panels operating street light posts using dust shields. A novel dust shield having the same width of the panel, and subtending an angle of 120° with the panel, is proposed for dust mitigation. Numerical simulations are carried out to evaluate the influence of the dust shield on dust accumulation over the panel’s surface. It is found that using a dust shield decreases the dust deposition rate by more than 44%. Moreover, extending the panel’s surface at the lower edge with an extension plate together with the dust shield decreases the dust deposition rate better than using a dust shield only. Also, the effect of adding an air gap between the shield and the added extension plate is investigated, and it is found that the air gap induces air drafts over the panel’s surface, which acts as an air barrier that obstructs the approach of dust particles to the panel’s surface. These drafts get stronger as the air gap thickness increases, accordingly, less particles deposit on the panel. Finally, it is found that using a dust shield with a length smaller than the panel’s length in addition to an extension plate together and increasing the thickness of the air gap is an effective and efficient solution for dust mitigation, such that the percentage decrease in the dust deposition rate that might be more than 88%.

## Introduction

Nowadays, the world energy consumption is increasing drastically. Non-renewable energy sources, i.e., fossil fuels, produce about 80% of the world’s energy consumption, thus leading to the rapid depletion of the natural resources (Rehfeldt et al. 2020). Therefore, it is important to decrease the use of traditional non-renewable energy resources and shift towards renewable energy sources; renewable energy is the most promising solution to the energy crisis (Wolniak and Skotnicka-Zasadzień 2022). Among the various types of renewable energy sources is the solar energy. One of the simplest methods of converting solar energy into electrical energy is using photovoltaic (PV) panels (Ahmed et al. 2022). Nowadays, most of the world’s plans and efforts are focused upon finding new ways to mitigate the factors which can reduce the efficiency of the PV panels. Among these factors is the soiling effect, which is caused by accumulation of dust on the panel’s surface (Shenouda et al. 2022a).

Dust accumulation on PV panels is given much concern nowadays especially in the MENA region. Several studies were done to investigate the influence of dust on the solar panel’s efficiency (Ekinci et al. 2022; Shenouda et al. 2022b). In a study conducted by Mustafa et al. (2020) in Jordan, it was found that dust accumulation on the panel’s surface has decreased the power output by 8.8% and the efficiency by 11.86%. In another study performed by Hamid et al. (2021) in Egypt, it has been found that the PV modules operational efficiency has decreased drastically by more than 50% after leaving the panel’s surface for 75 days without cleaning. While in Saudi Arabia, the efficiency reduction was 28% after 3 months of operation (Benghanem et al. 2018) and 50% after 6 months of operation without cleaning according to Adinoyi and Said (2013). In a study conducted by Dhaouadi et al. (2021), it was found that the panel’s efficiency was reduced by more than 36% due to dust accumulation after 8 weeks in United Arab Emirates.

It is clear from the previous literature review that dust accumulation over PV panels leads to a significant reduction in the output energy. Thus, in order to increase the PV panels’ energy production, cheap and alternative ways should be found in order to mitigate the factors that can reduce the efficiency of the solar panels. Many techniques were implemented to clean and mitigate dust, especially for ground mounted PV panels, such as manual and self-cleaning techniques (Shenouda et al. 2022c). However, these techniques might not be suitable for highly elevated PV panels. For example, manual cleaning techniques are not adequate in case of highly elevated PV modules, since it is risky to stand on a ladder for cleaning the panel. Moreover, the initial and maintenance costs in case of using autonomous robots and self-cleaning techniques are very high (Shenouda et al. 2022c; Eisa et al. 2023). Therefore, it is not feasible to apply such a technique for cleaning a single panel installed at an elevated height, i.e., light post. Passive cleaning techniques such as using super hydrophilic and super hydrophobic coatings were also developed and extensively applied. All coatings help in improving the performance of the PV panels to a certain limit (Syafiq et al. 2022; Hossain et al. 2022); however, their effect is dependent on the rainfalls, which is rare in the MENA region. Therefore, dust mitigation techniques using water are not feasible for the MENA region due to the scarcity of fresh water. A new dust mitigation technique was developed by Raillani et al. (2022), which uses a wind barrier that is installed on the ground in front of the PV panel, in order to obstruct the wind carrying the dust particles. The area of the barrier should be greater than the panel’s area in order to obstruct more dust. It was found that the amount of deposited dust particles on the panel’s surface can be reduced by 86% for large particles and 33% for small particles. However, this type of wind barriers is not applicable for PV panels mounted on light posts at elevated heights greater than 3 m. In such a condition, a wind barrier of height larger 3 m should be mounted for each light post which is not practical, especially in case of a bank of light posts.

Therefore, it is required to develop a dust mitigation technique that is not water dependent, has a low initial and running costs, in addition to being suitable to the small size of PV panels operating street light posts. The objective of this research is to study the influence of using a dust shield, which is mounted on the panel and subtending an angle of 120° with the panel, on the deposition of dust particles over the panel’s surface. The function of the dust shield is to obstruct the dust path, such that it minimizes the dust deposition rate on the panel’s surface. Afterwards, the effect of adding a plate, as an extension to the panel, together with the dust shield to act as a nozzle, is studied. Moreover, the effect of increasing the thickness of the air gap between the shield and the added extension plate is also studied. Finally, the combined effect of changing the length of the dust shield, along with increasing the air gap thickness on the dust deposition rate is also investigated.

## Numerical methodology

### Numerical scheme

The CFD package ANSYS FLUENT 18.1 is used to simulate air and dust flow around a PV panel and predict the dust particles behavior. The CFD package was used to solve the Navier–Stokes equations of airflow around the PV panel and the dust particles governing equations for dust motion. The Eulerian–Lagrangian approach was used to simulate the airflow and dust particles’ trajectories around the PV panel. The Eulerian approach was used to compute the continuous fluid phase which was assumed to be steady, incompressible 2D air flow, while the Lagrangian technique was adopted to predict particles transport in air.

### Air flow around a PV panel

The turbulent fluid flow is governed by the Reynolds averaged Navier–Stokes (RANS) equations that are based on the laws of the conservation of mass and momentum. The time-averaged mass and momentum equations for the turbulent airflow fields around the PV panel can be described by Eqs. (1) and (2) that governs the mean-velocity and pressure fields of incompressible turbulent flow (Tian and Ahmadi 2007).

1 $$\frac{\partial {\overline{u} }_{i}}{\partial {\overline{x} }_{i}}=0,i=\mathrm{1,2}$$

2 $$\frac{\partial {\overline{\mathrm{u}} }_{\mathrm{i}}}{\partial \mathrm{t}}+{\overline{\mathrm{u}} }_{\mathrm{j}}\frac{\partial {\overline{\mathrm{u}} }_{\mathrm{i}}}{\partial {\mathrm{x}}_{\mathrm{j}}}=-\frac{1}{\uprho }\frac{\partial \overline{\mathrm{p}}}{\partial {\mathrm{x} }_{\mathrm{i}}}+\frac{1}{\uprho }\frac{\partial }{\partial {\mathrm{x}}_{\mathrm{j}}}\left(\mathrm{v}\frac{\partial {\overline{\mathrm{u}} }_{\mathrm{i}}}{\partial {\mathrm{x}}_{\mathrm{j}}}-\uprho \overline{{\mathrm{u} }_{\mathrm{i}}^{\mathrm{^{\prime}}}{\mathrm{u}}_{\mathrm{j}}^{\mathrm{^{\prime}}}}\right)\mathrm{i},\mathrm{j}=\mathrm{1,2}$$

where $\rho$,$v$, ${u}_{i}$ and $\overline{p }$ are the fluid density, fluid kinematic viscosity, time-averaged velocity and pressure, respectively. The last term on the right-hand side of Eq. (2) represents the force due to turbulence per unit volume where $\rho \overline{{u }_{i}^{^{\prime}}{u}_{j}^{^{\prime}}}$ is the Reynolds stress tensor and ${\mathrm{u}}_{i}^{^{\prime}}$ represents the fluctuation velocity of the fluid. The Reynolds stresses make the number of unknowns higher than the number of RANS equations. Consequently, a turbulence model should be adopted as a closure to the equations in order to predict the turbulent air flow around the solar PV panel (Wilcox 2006). the numerical results for airflow around a panel performed by several studies (Karava et al. 2011; Lu and Zhao 2019) proved that the shear stress transport (SST) *k-ω* turbulence model has the best prediction results among different turbulence models. The transport governing equations of the (SST) *k-ω* turbulence model are addressed by Eqs. (3) and (4) (Tian and Ahmadi 2007),

3 $$\frac{\partial }{\partial t}\left(\rho \kappa \right)+\frac{\partial }{\partial {x}_{i}}\left(\rho \kappa {u}_{i}\right)=\frac{\partial }{\partial {x}_{j}}\left({\Gamma }_{\kappa }\frac{\partial \kappa }{\partial {x}_{j}}\right)+{G}_{\kappa }-{Y}_{\kappa }+{S}_{\kappa },$$

4 $$\frac{\partial }{\partial t}\left(\rho \omega \right)+\frac{\partial }{\partial {x}_{i}}\left(\rho \omega {u}_{i}\right)=\frac{\partial }{\partial {x}_{j}}\left({\Gamma }_{\omega }\frac{\partial \omega }{\partial {x}_{j}}\right)+{G}_{\omega }-{Y}_{\omega }+{D}_{\omega }+{S}_{\omega }.$$

where *G*_*k*_ (m^2^/s^2^) and *G*_*ω*_ (s^−1^) are the generation of turbulent kinetic energy *k* and the generation of *ω*, respectively. The effective diffusivity of *k* and *ω* are given by *Γ*_*k*_ and *Γ*_*ω*_, respectively. *Y*_*k*_ and *Y*_*ω*_ are the dissipation rates of *k* and *ω*, respectively, due to turbulence. *D*_*ω*_ stands for the cross-diffusion term. *S*_*k*_ and *S*_*ω*_ are user defined source terms respectively and both are taken as zero.

### Boundary conditions

In this study, the initial wind inlet velocity is 4 m/s which is the average wind speed in Egypt (Anon). The initial pressure is equal to the standard atmospheric pressure in the whole computational domain. The input data is the boundary conditions in the computational domain. A turbulent boundary layer is obtained when the Reynolds number *Re* = *ρvL/μ* becomes larger than 100,000. In this study, air density, *ρ,* is 1.225 kg/m^3^, air velocity, *v,* is 4 m/s, the representative length (*L*) is 1.2 m, and dynamic viscosity of air, *μ,* is 1.79 × 10^−5^ kg/m.s. Accordingly, a Reynold’s number of 328,601 is achieved indicating a turbulent flow around the panel. The no-slip boundary condition was imposed on all the walls and the roughness model used for the wall roughness is the standard model. The outflow boundary condition was applied on the outlet, while the symmetry boundary condition was applied on the upper boundary of the computational domain according to (Tian and Ahmadi 2007; Wilcox 2006).

### Dust deposition behavior

In the present study, dust particles were considered as a discrete phase and the trajectory of each dust particle was tracked through solving the particle dynamic equation, using the effective DPM model. This model is most suitable for modeling a two-phase flow problem involving a single continuous discrete phase having a negligible volume fraction of less than 12% (Ahmad et al. 2015). The influence of dust motion on the fluid flow field and particle–particle interactions can be neglected since dust-laden air flow is dilute enough.

The governing equation of the dust motion is described by Eq. (5) as follows (Ahmad et al. 2015).

5 $${\mathrm{m}}_{\mathrm{p}}\frac{\mathrm{d}{\overrightarrow{\mathrm{u}}}_{\mathrm{p}}}{\mathrm{dt}}=\frac{1}{2}{\mathrm{C}}_{\mathrm{D}}{\mathrm{A}}_{\mathrm{p}}\uprho \left|\overrightarrow{\mathrm{u}}-{\overrightarrow{\mathrm{u}}}_{\mathrm{p}}\right|\left(\overrightarrow{\mathrm{u}}-{\overrightarrow{\mathrm{u}}}_{\mathrm{p}}\right)+{\mathrm{m}}_{\mathrm{p}}\overrightarrow{\mathrm{g}}-\uprho {\mathrm{V}}_{\mathrm{p}}\overrightarrow{\mathrm{g}}+\overrightarrow{\mathrm{F}}$$

The first term in the right-hand side of Eq. (5) represents the drag force where the drag coefficient is represented by ${C}_{D}$, the particle cross-sectional area is represented by ${A}_{p}$ and ${\overrightarrow{u}}_{p}$ is the velocity of the dust particle. The second term, ${m}_{p}\overrightarrow{g}$, represents the weight of the particle, where ${m}_{p}$ is the particle mass and the third term, $\rho {V}_{p}\overrightarrow{g}$, represents the buoyancy force, where ${V}_{p}$ is the volume of the dust particle. The last term of the right-hand side of Eq. (5), $\overrightarrow{F}$, represents the Saffman’s lift force which is the lift due to shear. On the other hand, the effect of the pressure gradient, the virtual mass and the Basset forces on the particle dynamics were neglected as compared to the external forces, since the ratio of air density to particle density is so small (0.00043) (Ahmad et al. 2015). The turbulent dispersion of dust particles due to airflow velocity fluctuations is modeled by discrete random walk (DRW) model. The DRW model is a stochastic approach used to improve the prediction accuracy (Ahmad et al. 2015). Equations (1) to (5) are used to solve the air-dust two phase flow field in the corresponding computational domain. Spherical dust particles with a uniform spatial distribution were injected at the inlet within the computational domain after the simulations of the airflow has converged. The aim of the present study is to track the number of deposited dust particles on the panel’s surface. Therefore, it is assumed that all the particles that impact the surface of the PV panel will be deposited on the surface without re-suspension. Accordingly, the trap function was adopted in fluent solver. While, for the rest of the boundary conditions, the escape boundary condition is enabled.

### Solution strategy

The finite volume method (FVM) was used to solve the conservation laws of mass, and momentum equations for the turbulent airflow fields. The coupled scheme was applied to decouple the pressure and the velocity fields. The pressure was discretized by second-order scheme. The momentum, turbulent kinetic energy and the specific dissipation rate were discretized by second order upwind. The Runge–Kutta method was adopted to solve the dust particle motion equations. The solution was considered to be converged when the residual mean square error (RMSE) value reaches 10^−6^. The numerical solutions of fluid flow problems using CFD solvers are approximate solutions since the numerical simulations are subject to various uncertainties due to the sources of error. These errors fall into two basic categories: namely acknowledged errors and unacknowledged errors. The acknowledged errors consist of modelling, discretization, convergence, computer round-off and truncation errors while the unacknowledged errors are code and usage errors (Sadrehaghighi 2022).

## Model geometry and case description

**Fig. 1:**
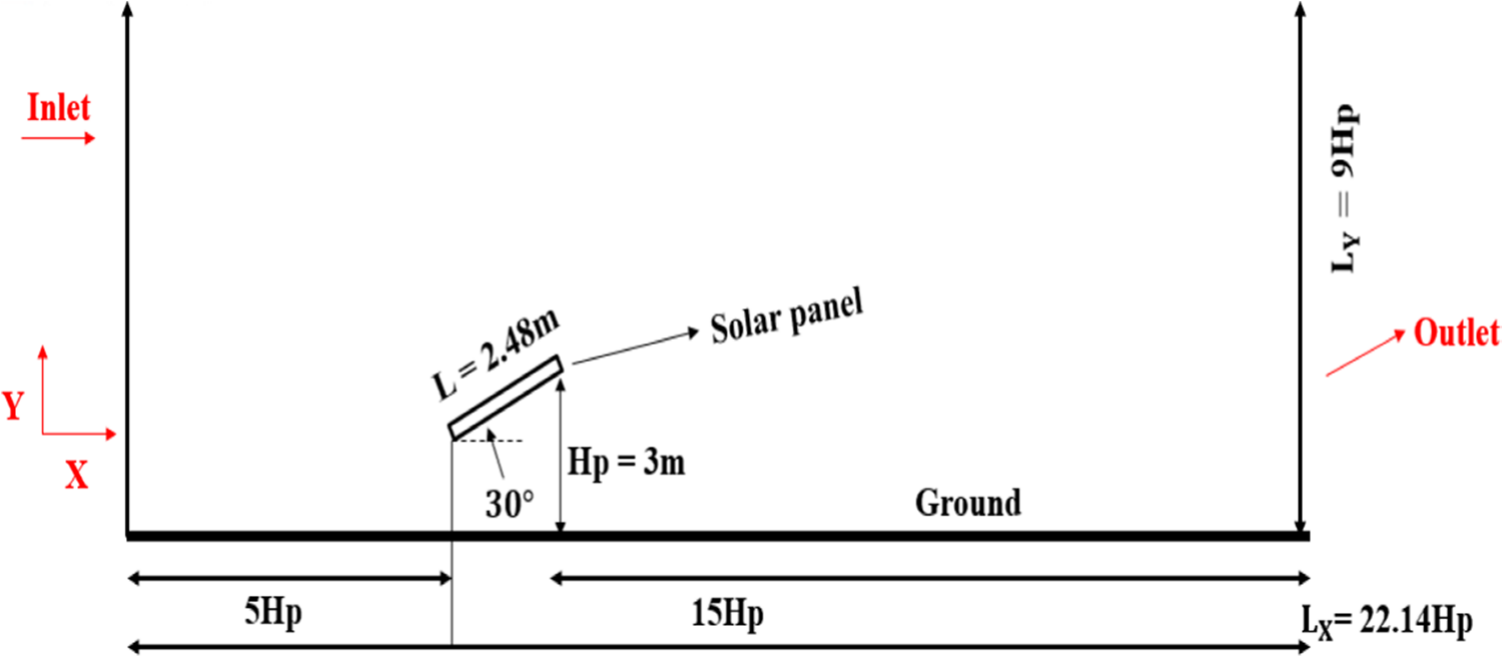
A schematic drawing of a PV panel mounted on a light post

The simplified physical model used for the present study is shown in Fig. 1. The PV panel was mounted on a light post. The PV panel is inclined at an angle of 30° above the horizontal. The PV panel is equipped with a dust shield. The dust shield has the same dimensions as the PV panel, and the angle between the PV panel and the dust shield is 120°, as shown in Fig. 1. An angle of 120° was selected based on an experimental study performed by (Eisa et al. 2023), in order to avoid the shading that can occur due to the dust shield where shading can occur if the angle is less than 120°. On the other hand, if the angle is more than 120°, the influence of the dust shield on the dust deposition will be less, since the length of the shield that obstructs the wind is decreased. The computational domain used in the present study was designed according to the experimental wind tunnel setup conducted by Tominaga et al. (2015). In this study, the computational domain was designed as *L*_*x*_ = 22.4*H*_*P*_ long and *L*_*y*_ = 9*H*_*P*_ high, where *H*_*P*_ is the height of the PV panel from the ground, as shown in Fig. 1. The panel’s height, *H*_*P*_*,* and length *L* are 3 m, and 2.48 m, respectively. In this study, the distance between the inflow, i.e., air inlet boundary, and the PV panel was taken as 5*H*_*P*_, and the distance from the PV panel to the outflow, i.e., outlet boundary, was 15*H*_*P*_, for the wake flow redevelopment. The aforementioned distances were widely used in the numerical simulations of wind flow over PV panels (Jubayer and Hangan 2014). After the airflow fields reached the convergence with a residual of 10^−6^, spherical dust particles with uniform size distribution were released from the domain inlet, and deposition of particles on the panel’s surface was then computed. The motion of the dust particles was modeled by the discrete particle model (DPM). The dust type and the number of deposited dust particles are important parameters that greatly affect the PV performance. The dust type was assumed as calcium carbonate whose density is 2800 kg/m^3^. A group of 10,000 dust particles was injected in the simulations. Each injection was tracked 10 times to obtain statistically valid and accurate results for tracking. The dust size range in the present study was chosen from 35 μm up to 90 μm.

## Grid independence study and numerical validation

**Fig. 2:**
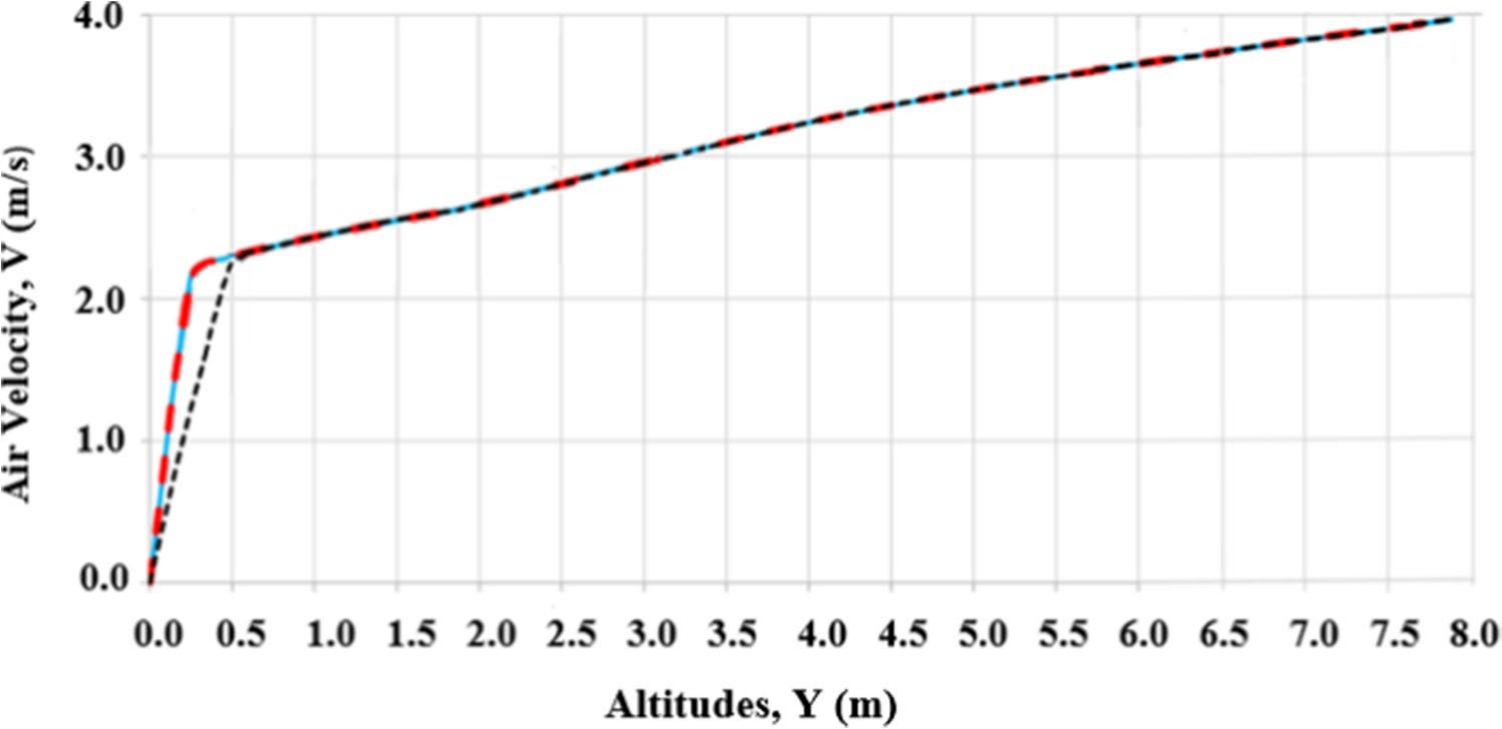
Air velocity profiles at *X* = 8 m from the inlet for different grids at different altitudes

The grid independence test was performed to determine the minimum mesh size that gives the best results. Four grids were created, including a coarse mesh with 28,231 cells, a medium mesh with 57,145 cells, a fine mesh with 78,120 cells, and a very fine mesh with 104,134 cells. The airflow velocity profiles are compared for the different cells, at a distance of 8 m from the inlet as shown in Fig. 2. It was found that the results of the medium, fine, and very fine meshes have almost the same velocity profile. However, the velocity profile of the coarse mesh is somehow different from those of the other meshes. Accordingly, the fine grid was adopted in the present study. The velocity profile of the very fine mesh is coincident with the medium and fine grids velocity profiles; therefore, it is not included in Fig. 2.

**Fig. 3:**
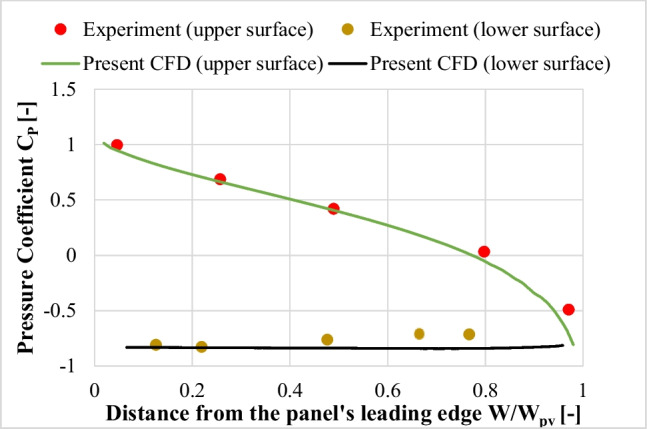
Validation by comparing the mean pressure coefficient, *C*_*P*_, of the experimental results and the CFD model

To validate the airflow fields, the mean pressure coefficient (*C*_*P*_) profiles are calculated on both the upper and lower surfaces of the panel (Dagher and Kandil. 2022). The *C*_*P*_ was calculated against the distance from the panel’s leading edge *W/W*_*PV*_; *W* represents the distance measured along the solar panel width from the leading edge while *W*_*PV*_ is the solar panel width. The results obtained from the numerical simulation model was compared with the experimental results conducted by Abiola-Ogedengbe et al. (2015), as shown in Fig. 3. The experiments were conducted in an open return wind tunnel, which has a length of 33 m, a width of 2.4 m and a height varying from 1.5 m at the entrance to 2.15 m at the test area. The tested PV panel was mounted at an angle of 30° above the horizontal with dimensions of 0.72 m × 0.24 m × 0.17 m. The Reynolds number of the performed experiment was 369,553 at an air velocity of 15 m/s. It was found that the maximum deviation of the mean *C*_*P*_ between the numerical simulations and the experimental measurements is about 1.04%. Therefore, the proposed numerical model is able to predict the airflow fields and the behavior of deposited dust particles accurately.

## Numerical simulations

**Fig. 4:**
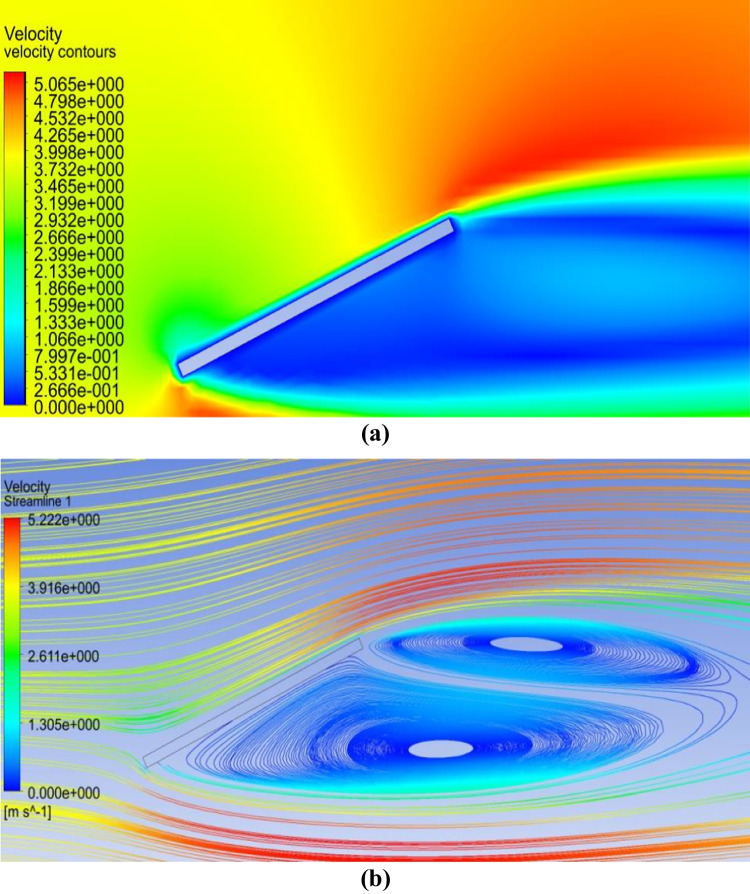
**a **Air velocity contours and** b** air velocity streamlines around a PV panel

The airflow field around a single PV panel is shown in Fig. 4. The velocity contours and streamlines around the PV panel are displayed in Fig. 4a and b, respectively. It can be observed from Fig. 4 that the flow field is blocked due to the presence of the PV panel as an obstacle. Moreover, it can be noted that there is a velocity variation around the panel where the velocity increases along the panel’s upper surface, while the airflow is almost stagnant at the backside of the panel. The airflow field has a significant effect on dust motions and deposition behaviors. Therefore, in case of the presence of dust particles in the airflow, the dust particles will accumulate on the surface since the dust particles follow the velocity streamlines that reach the panel’s surface. The dust deposition rate over a panel without shield is taken as a reference for comparison with the developed dust mitigation techniques.

Four sets of numerical simulations were conducted to infer the influence of the following parameters on dust accumulation over PV panels: (1) dust shield, (2) extending the length of the panel by adding an extension plate in the presence of the dust shield to act together as a nozzle in case of an air gap, (3) increasing the air gap between the panel and the dust shield, as well as (4) changing the length of the shield. The performed simulations are summarized in Table 1.

**Table 1:** Tested variables in the performed numerical simulations

Numerical simulation	Variable			
	Shield	Extension plate	Air gap between shield and panel	Shield length
(1)	** × **	-	-	-
(2)	** × **	** × **	-	-
(3)	** × **	** × **	** × **	-
(4)	** × **	** × **	** × **	** × **

## Results and discussions

### Airflow around a PV panel with a dust shield

**Fig. 5:**
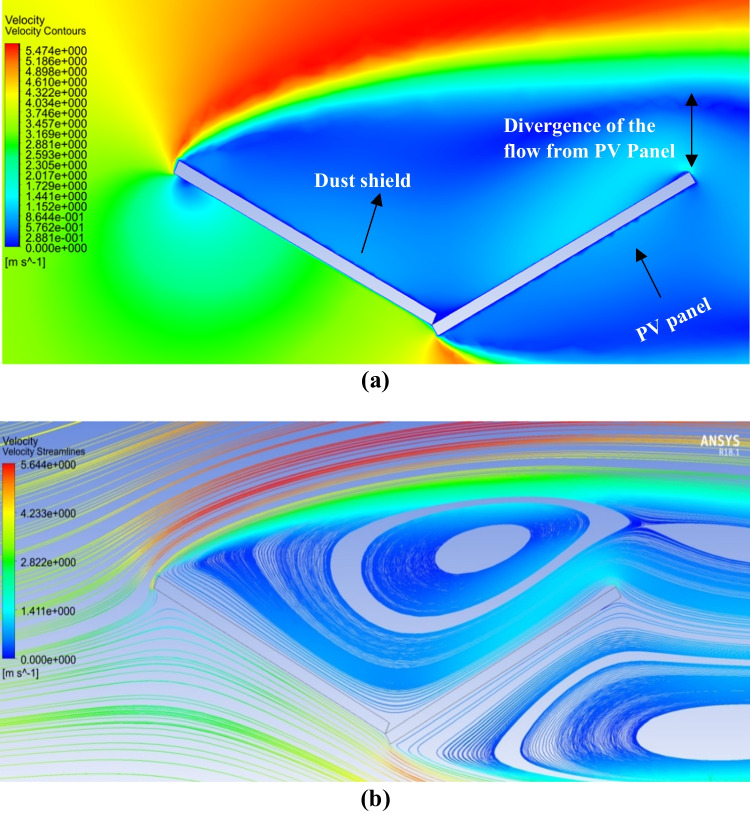
**a **Air velocity contours and **b** air velocity streamlines around a PV panel with a dust shield

Numerical simulations were carried out to evaluate the influence of using a dust shield added to the PV panel. The velocity contours and streamlines of the airflow around a PV panel with a dust shield are shown in Fig. 5. It is clear from the velocity contours that the dust shield obstructs the airflow path such that the path of the airflow is diverted along the upper tip of the shield and moves away from the panel’s surface, as shown in Fig. 5a. Accordingly, the dust particles follow this diverted flow having high velocity; thus, the chance of dust deposition on the panel’s surface is decreased. The velocity magnitude is increasing along the upper tip of the dust shield, i.e., away from the area surrounding the surface of the panel, while the area between the shield and the panel is a stagnant area where the velocity of the airflow is nearly 0 m/s due to obstruction of the airflow by the dust shield. Thus, a vortex motion occurs due to the change in velocity, as shown in Fig. 5b. Therefore, the chance of dust deposition on the panel’s surface is reduced much more than that in the case of no shield.

#### Dust deposition rates for different dust sizes in case of a PV panel with dust shield and no shield

Dust deposition rate on PV panels is influenced by many factors, such as the velocity, inertia and gravitational effect of dust particles, the airflow, and the turbulent kinetic energy distribution around the PV panel. The effect of the dust particle’s size on the deposition rate in cases of a PV panel with shield and no shield was analyzed using CFD simulations. The dust deposition rate *λ* is defined as follows:

6 $$\lambda =\frac{{N}_{d}}{{N}_{p}}\times 100\%.$$

where *N*_*P*_ and *N*_*d*_ are the number of injected and deposited dust particles on the panel’s surface, respectively. The dust deposition rates on the panel’s surface, in cases of shield and no shield for different dust sizes, are presented in Table 2. It is clear that increasing the size of the dust particle has an obvious impact on the dust deposition rate; as the size of the particle increases, the dust deposition rate increases, as shown in Table 2. This is attributed to the influence of inertia and gravity, which are the dominant factors. Larger-sized particles are deposited on the panel’s surface more than smaller-sized particles that are rather influenced by the Brownian motion and often remain floating in the air without deposition. The percentage drop in the dust deposition rate due to the added shield as compared to the no shield case, %*Δλ*, is calculated based on the following equation:

7 $$\%\Delta \lambda =\frac{{\lambda }_{no-shield}-{\lambda }_{shield}}{{\lambda }_{no-shield}}\times 100\%$$

where *λ*_*no-shield*_, and *λ*_*shield*_ are the dust deposition rates in cases of a PV panel with no shield and an added shield. The percentage drop in the dust deposition rate due to the added shield is presented in Table 2, and it can be seen that using a dust shield reduces the dust deposition rate by almost 44 to 65%, which indicates that using a dust shield is an effective technique for dust mitigation of PV panels.

**Table 2:** Dust deposition rate in case of shield and no shield for different dust sizes

	Dust deposition rate, λ		
Particle size	No dust shield λ_no-shield_	dust shield λ_shield_	%Decrease in deposition rate, %Δλ
35 μm	0.39%	0.16%	59%
40 μm	0.45%	0.18%	60%
45 μm	0.54%	0.20%	62.5%
50 μm	0.66%	0.25%	63.5%
55 μm	0.76%	0.28%	65%
60 μm	0.89%	0.34%	62%
75 μm	1.18%	0.55%	54%
80 μm	1.30%	0.65%	52%
90 μm	1.53%	0.86%	44%

### Effect of adding an extension plate to the PV panel on the dust deposition rate

**Fig. 6:**
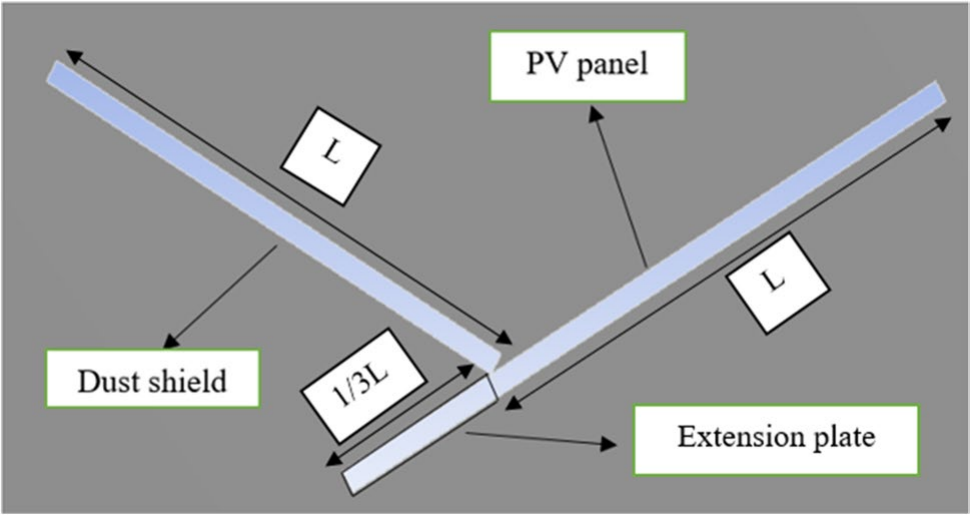
A new geometry consisting of an added plate as an extension to the panel

**Fig. 7:**
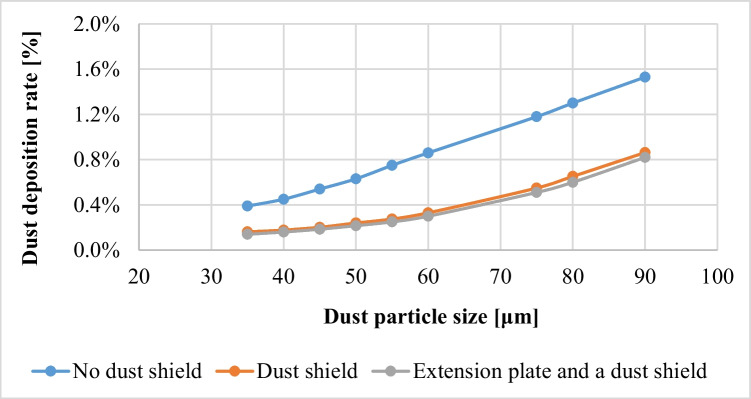
Dust deposition rate in cases of **a** a no shield, **b** a dust shield, and **c** an extension plate with a dust shield for different dust sizes

**Fig. 8:**
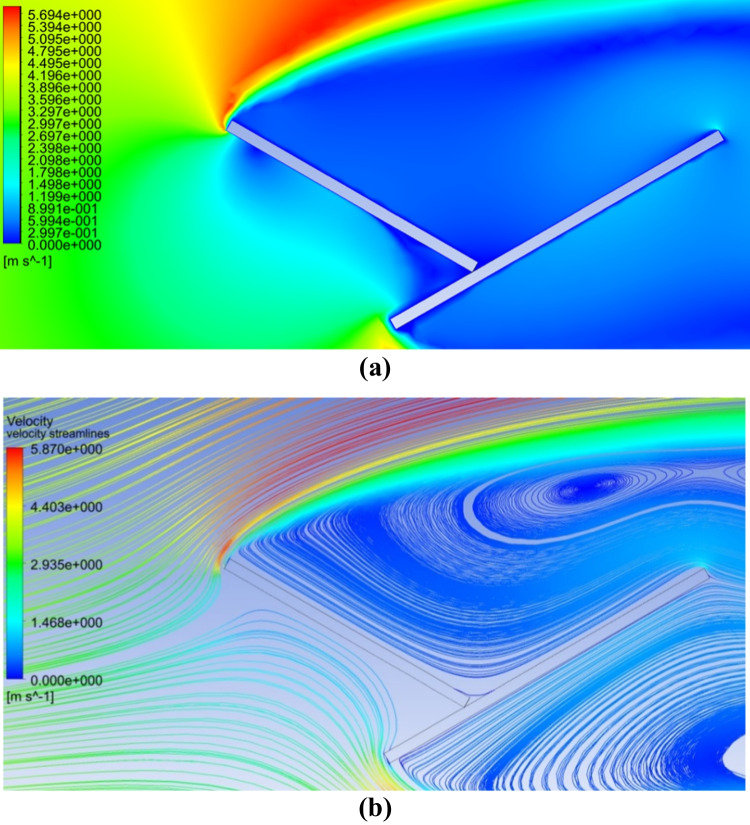
**a **Air velocity contours and** b** air velocity streamlines in case of adding an extension plate and a dust shield

The effect of adding an extension plate together with the dust shield on the dust deposition rate on the panel’s surface is presented in this section. The added plate is an extension of the PV panel, as shown in Fig. 6, and the length of the extension plate is taken as one third of the panel’s total length, *L*. The dust deposition rates in cases of no shield, a dust shield, and an extension plate together with a dust shield are presented in Fig. 7. It was found that adding an extension plate decreases the number of deposited particles as compared to that in the case of no shield. The velocity contours and streamlines of the airflow around a PV panel with a dust shield, in the presence of an extension plate, are shown in Fig. 8. It is noted from Fig. 8 that the airflow field is very similar to that in the case of using a dust shield shown in Fig. 5, which explains the marginal difference in the deposition rate between the two cases as shown in Fig. 7.

### Effect of increasing the size of the air gap between the shield and the panel

**Fig. 9:**
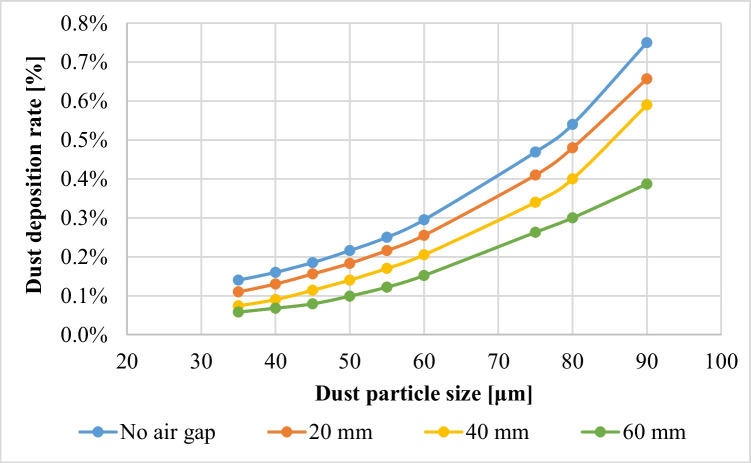
Dust deposition rate on the panel’s surface in case of adding an extension plate and air gaps for different dust particle sizes

**Fig. 10:**
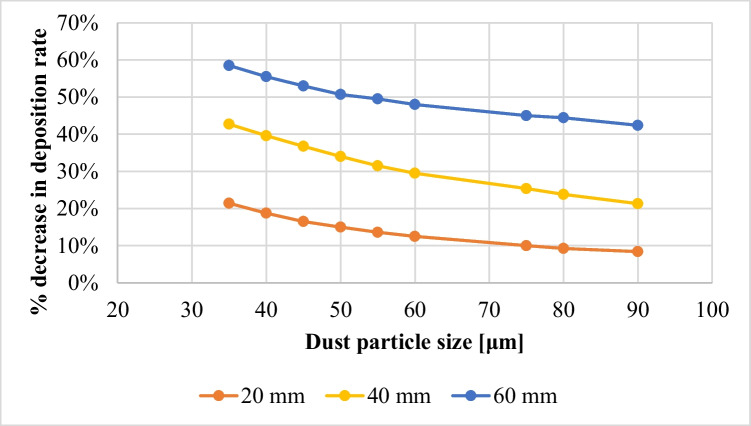
Percentage decrease in the dust deposition rate, *%*Δλ, in case of an added extension plate and increasing air gaps for different dust sizes (percentage decrease in the dust deposition rate, $\%\Delta\lambda=\frac{\lambda_{\mathrm{no}\;air\;gap}-\lambda_{air\;gap}}{\lambda_{\mathrm{no}\;air\;gap}}\times100$

**Fig. 11:**
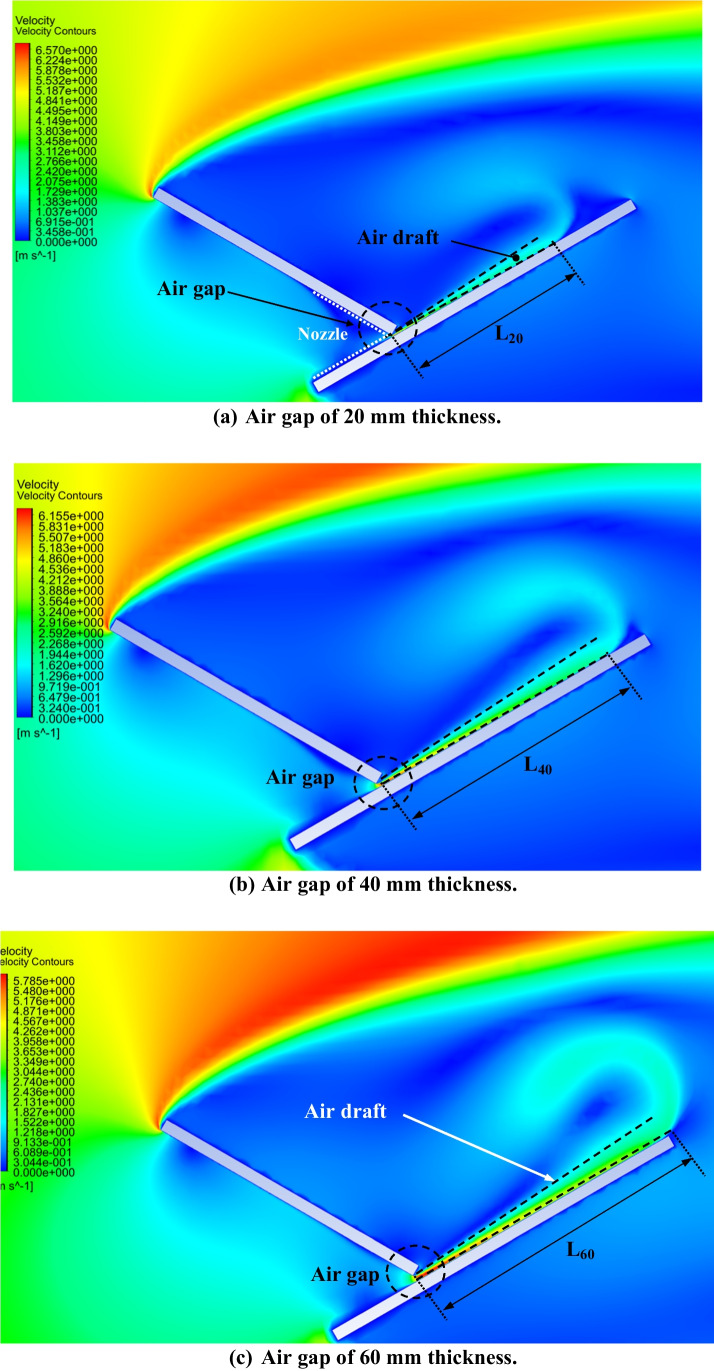
Air velocity contours around the new geometry as a function of the air gap size: **a** 20 mm, **b** 40 mm, and **c** 60 mm. L is the swept length by the air draft over the panel’s surface

The effect of increasing the air gap size between the PV panel and the dust shield on the dust deposition rate, in the presence of the extension plate, is presented in Fig. 9, where the thicknesses of the air gaps are 20, 40, and 60 mm, as indicated. It is found that the dust deposition rate decreases with increasing the air gap size, such that the maximum deposition rate occurs at the minimum air gap, while the minimum deposition rate occurs at the maximum air gap, i.e., 60 mm. The percentage decrease in dust deposition rate on the panel’s surface in the presence of an extension plate, as a function of the different air gaps and dust sizes, is presented in Fig. 10. The dust deposition rates in cases of particles size of 35 μm and 60 mm air gap decrease by 58.5% as compared to the case of no air gap, while in case of an air gap of 40 mm, it decreases by 43%, and in case of an air gap of 20 mm, it decreases by 21.56%. This is due to the increase in the air drafts as the size of the air gap increases, which acts as an air barrier that decreases the approach of dust particles to the panel’s surface. The size of the air barrier increases as the air gap increases, consequently, less particles approach the panel’s surface and accordingly, the dust deposition rate decreases. It can be concluded that introducing an extension plate in addition to a dust shield helps in reducing the dust deposition rate as compared to applying a dust shield only. In addition to that, increasing the air gap between both the shield and the panel decreases the dust deposition rate of the panel’s surface because of the introduced air drafts. The air velocity contours in case of using an extension plate in addition to a dust shield and increasing air gaps are presented in Fig. 11. The influence of the air gap on dust deposition over the PV panel was investigated for different dust sizes. The added extension plate to the PV panel together with the dust shield act as an air nozzle, such that the air velocity increases as the air enters the nozzle until the nozzle throat, i.e., the air gap, where the fluid velocity at the nozzle throat is maximum, as can be seen in Fig. 11. At the air gap, there is a pressure loss, so increasing the size of the air gap minimizes the pressure losses, consequently, increases the exit air velocity from the air gap. The air passing out of the nozzle keeps intact with the PV panel’s surface and does not divert from the panel’s surface, which is known as the Coanda effect (Lee et al. 2021; Ahmed 2020; Shakouchi and Fukushima 2022), and such a performance assists in removing the deposited dust particles. Increasing the air gap size between the shield and the panel to a certain extent induces more kinetic air drafts; thus, more area of the panel’s surface is swept by the air draft as can be seen by comparing Fig. 11 a, b, and c. Consequently, the flow of dust is diverted from approaching the panel’s surface; therefore, the dust deposition rate is reduced by obstructing the dust particles.

### Effect of changing the length of the dust shield

#### Dust deposition rate in case of changing the shield length in case of no air gap

**Fig. 12:**
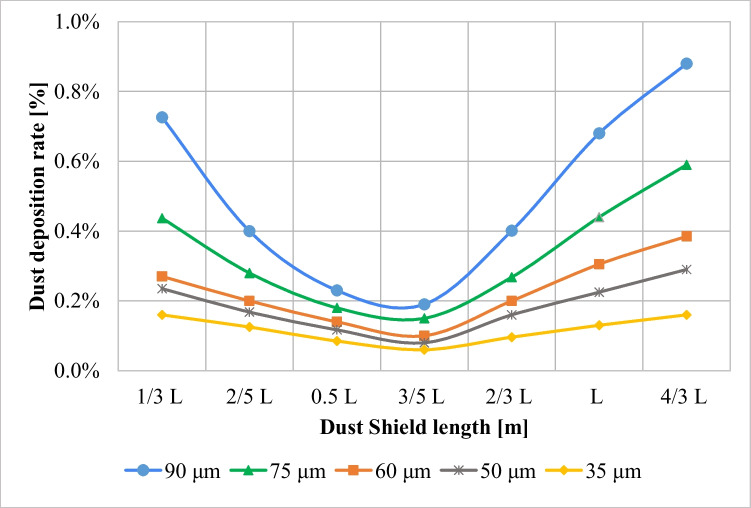
Dust deposition rates in case of different dust shield lengths, i.e., 4/3–1/3 L, at no air gap for different dust sizes, where L is the panel’s length

**Fig. 13:**
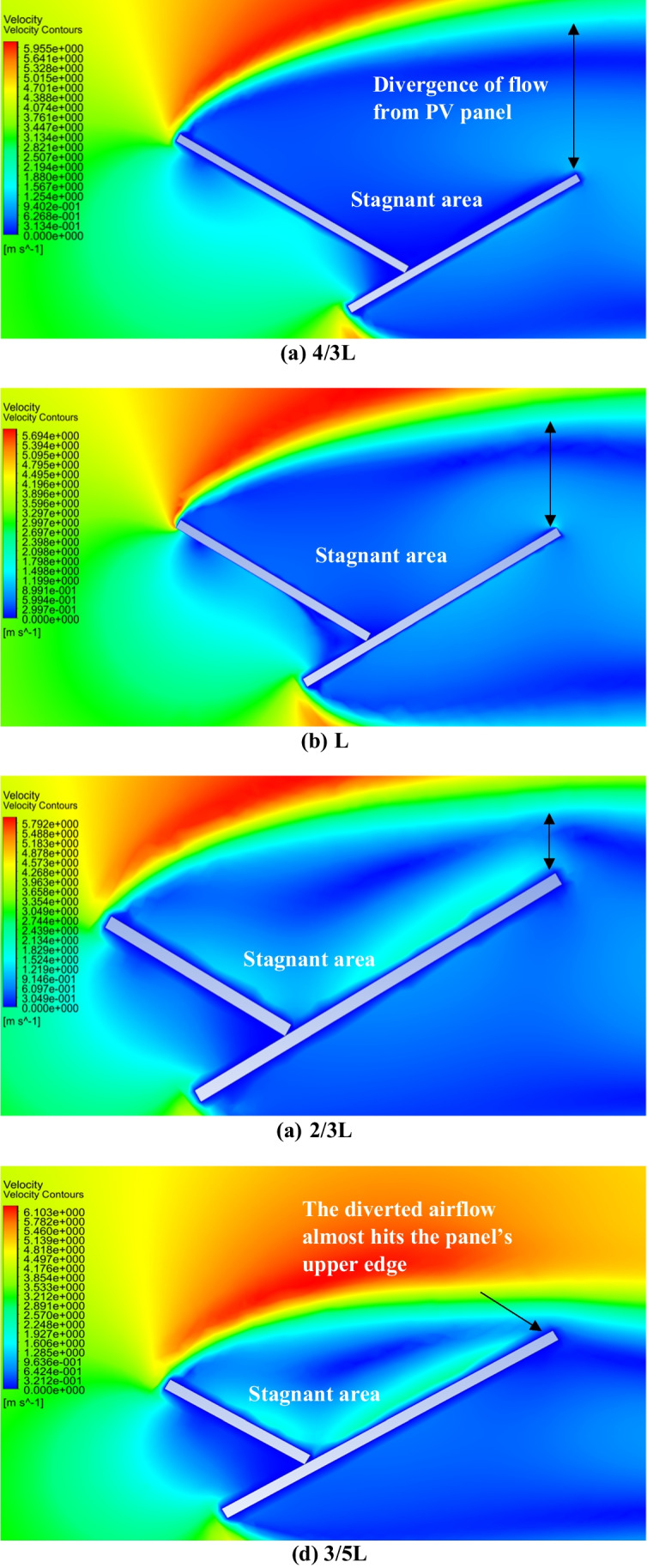
Air velocity contours of the new geometry in case of no air gap for different shield lengths, i.e., **a** 4/3 L, **b** L, **c** 2/3 L, and **d** 3/5 L, where L is the panel’s length

The introduction of a dust shield has proven to be an effective dust mitigation technique for decreasing the deposition of dust particles especially if an air nozzle is created between the panel and the shield. In this section, the influence of the dust shield length is investigated and the results are discussed. Different dust shields with lengths 4/3 L, L, 2/3 L, 3/5 L, 0.5 L, 2/5 L, and 1/3 L have been examined. The dust deposition rates, in case of different dust shield lengths at no air gap for different dust sizes, are shown in Fig. 12. It can be seen from Fig. 12 that increasing the dust shield length from 1/3 to 3/5 L decreases the dust deposition rate, for different dust sizes, after which increasing the shield length above 3/5 L increases the dust deposition rate. Therefore, it can be concluded that 3/5 L is the optimum point at which the dust deposition rate reaches its minimum value, after which it begins to increase again from 3/5 to 4/3 L. The air velocity contours for the new geometry in case of no air gap for different dust shield lengths, (a) 4/3 L, (b) L, (c) 2/3 L, and (d) 3/5 L are presented in Fig. 13. The reason behind the increase in the dust deposition rate as the shield length increases is the increase in the size of the stagnant area behind the shield. Increasing the size of the stagnant area between the shield and the panel increases the possibility of accumulation of more dust particles in this area. On the other hand, as the dust shield decreases, the dust accumulation rate decreases since the stagnant area size decreases. However, there is an optimum shield length after which the dust deposition rate begin to increase. This optimum length was found to be 3/5 L. This is because decreasing the shield length below a certain limit causes the divergence of the incoming air towards the panel’s surface as shown in Fig. 13. Consequently, the possibility of particles’ accumulation in the area between the panel and the shield increases.

#### Dust deposition rate in cases of varying the dust shield length in addition to increasing the air gap thickness

**Fig. 14:**
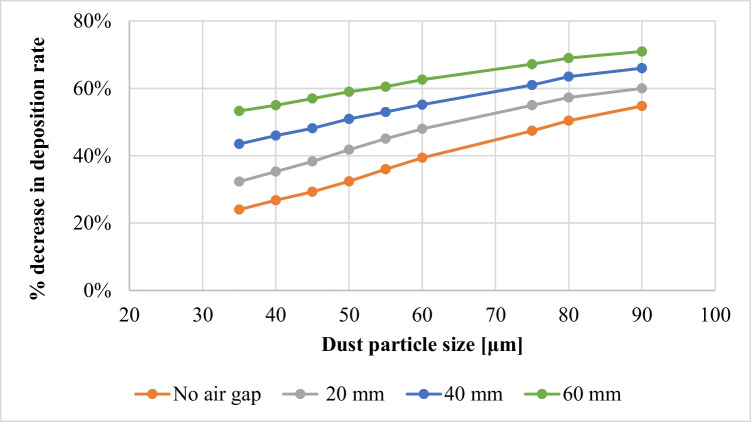
Percentage decrease in the dust deposition rate, *%*Δλ, in case of decreasing the shield length from 4/3 to 2/3 L in the presence of an extension plate for air gaps and different dust sizes

**Fig. 15:**
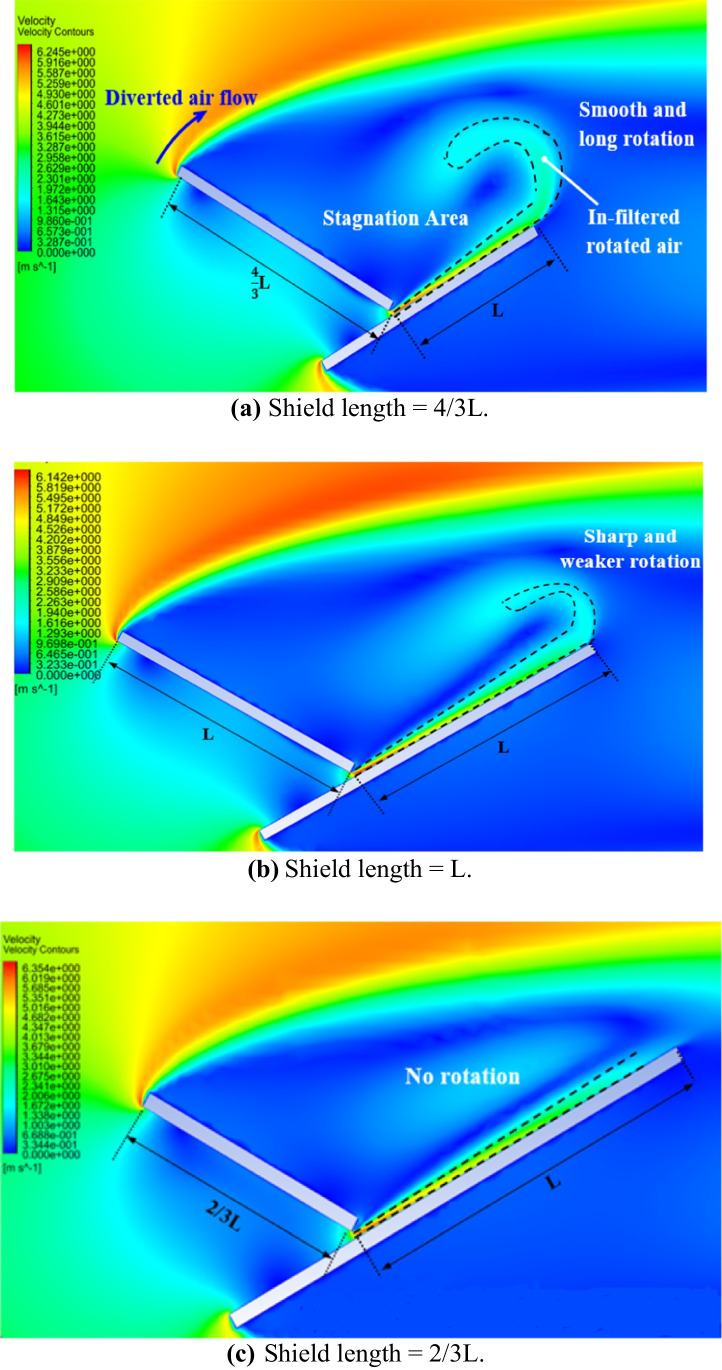
Air velocity contours of the new geometry in case of 60 mm air gap for different shield lengths, i.e., **a** 4/3 L, **b** L, and **c** 2/3 L, where L is the panel’s length

The dust deposition rates in cases of varying the shield length for different dust sizes and air gaps are shown in Table 3. It is found that decreasing the dust shield length to 2/3 L decreases the dust deposition rate, while increasing the dust shield length to 4/3 L increases the dust deposition rate as compared to the reference case where the length of the dust shield is L in case of the presence of an air gap. For example, the dust deposition rates in case of a dust particle size of 35 μm at an air gap of 60 mm, and dust shield lengths of 4/3 L, L, and 2/3 L, are 0.07%, 0.06% and 0.04%, respectively. On the other hand, the dust deposition rates in case of a dust particle size 90 μm at an air gap thickness of 60 mm, and dust shield lengths of 4/3 L, L, and 2/3 L, are 0.62%, 0.39%, and 0.18%, respectively. The percentage decrease in dust deposition rate in case of decreasing the dust shield length from 4/3 to 2/3 L, for different air gaps and different dust sizes, is shown in Fig. 14. For example, the dust deposition rate in case of the shortest shield of length 2/3 L, at no air gap for different particle sizes, decreases by 25–55% as compared to the case of shield of length 4/3 L. While the percentage decrease in dust deposition rate increases to 32–60% at an air gap size of 20 mm. Moreover, increasing the air gap thickness to 40 mm changes the percentage decrease from 43.5 to 66%. Finally, the maximum percentage decrease in dust deposition rate is achieved in case of 60 mm air gap where the dust deposition rate decreases by 53–71%. Figure 15 represents the air velocity contours of the new geometry in the presence of an air gap for different shield lengths of (a) 4/3L, (b) L, and (c) 2/3 L. The rotation of the in-filtered air, at the edge of the PV panel, is emphasized by the dotted line in Fig. 15, and it can be seen that it is proportional to the shield’s length. The rotation of the in-filtered air is large and smoothly rotating in case of the longest shield, as can be seen in Fig. 15a, and it diminishes with the shortest shield, as can be seen in Fig. 15c. In case of the shortest shield, the rotated in-filtered air is obstructed by the diverted airflow coming from the top edge of the dust shield, consequently, no vortices are obtained. On the other hand, as the shield’s length increases, the influence of the diverted flow on the in-filtered flow decreases, i.e., the chance of obstructing the rotation of the in-filtered flow by the diverted flow is getting less. Consequently, stronger rotations of the in-filtered air and more vortices are generated. Accordingly, the possibility of dust accumulation in the area between the panel and the shield increases. Therefore, it can be inferred that decreasing the length of the shield decreases the dust deposition rate.

**Table 3:** Dust deposition rates in case of different shield lengths for different air gaps and different dust sizes

	Dust deposition rate				
Particle size	Shield length	No gap	Gap 20 mm	Gap 40 mm	Gap 60 mm
35 μm	4/3 L L 2/3 L	0.15% 0.14% 0.11%	0.11% 0.10% 0.93%	0.09% 0.07% 0.05%	0.07% 0.06% 0.04%
40 μm	4/3 L L 2/3 L	0.18% 0.16% 0.13%	0.14% 0.13% 0.09%	0.10% 0.09% 0.06%	0.09% 0.07% 0.04%
45 μm	4/3 L L 2/3 L	0.21% 0.19% 0.17%	0.18% 0.16% 0.11%	0.14% 0.12% 0.08%	0.10% 0.08% 0.05%
50 μm	4/3 L L 2/3 L	0.26% 0.22% 0.18%	0.24% 0.19% 0.12%	0.19% 0.15% 0.09%	0.13% 0.10% 0.05%
55 μm	4/3 L L 2/3 L	0.31% 0.25% 0.20%	0.27% 0.22% 0.14%	0.21% 0.17% 0.10%	0.17% 0.13% 0.07%
60 μm	4/3 L L 2/3 L	0.37% 0.28% 0.22%	0.30% 0.25% 0.14%	0.24% 0.22% 0.11%	0.20% 0.15% 0.07%
75 μm	4/3 L L 2/3 L	0.53% 0.47% 0.27%	0.52% 0.42% 0.19%	0.44% 0.37% 0.16%	0.34% 0.27% 0.10%
80 μm	4/3 L L 2/3 L	0.62% 0.54% 0.32%	0.60% 0.49% 0.22%	0.52% 0.41% 0.16%	0.43% 0.36% 0.13%
90 μm	4/3 L L 2/3 L	0.82% 0.74% 0.40%	0.75% 0.66% 0.30%	0.69% 0.61% 0.22%	0.62% 0.39% 0.18%

### Discussion of results

**Fig. 16:**
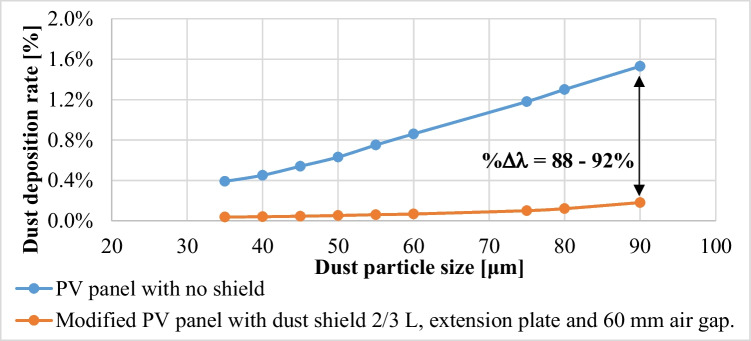
Percentage decrease in the dust deposition rate over a PV panel due to adding a dust shield of length 2/3 L, an extension plate, and an air gap thickness of 60 mm

Based on the presented results, it can be concluded that adding (a) a dust shield of length 2/3 L, (b) extending the surface of the panel, and (c) having an air gap thickness of 60 mm between the panel and the shield decreases the dust deposition rate in a much better way, as compared to a PV panel with no shield, as illustrated in Fig. 16. For example, in case of particle size of 35 μm, the dust deposition rate is 0.39% for a PV panel with no shield, while it is 0.04% in case of the modified panel, so it can be concluded that there is a 90% decrease in the dust deposition rate. Also, in case of particles’ size of 90 μm, the dust deposition rate is 1.53% for a PV panel with no shield, while it is 0.18% in case of the modified panel, which corresponds to 88% decrease in the dust deposition rate. It can be seen that the percentage decrease in the dust deposition rate is between 88 and 92%, as shown in Fig. 16. This proves that using a dust shield shorter than the panel and extending the surface of panel together with having an air gap between the panel and the shield is an effective solution for dust mitigation. Also, the induced air draft through the air gap between the dust shield and the PV panel could assist in cooling the PV panel and preserve the efficiency of the panel especially in hot environments.

The influence of different dust mitigation techniques on the dust deposition rate at a particle size of 90 μm is presented in Table 4. It can be concluded that adding a dust shield has a good impact on the deposition rate, such that the deposition rate has decreased in comparison to the no shield case by 44%, and creating an air nozzle together with the dust shield has significantly decreased the deposition rate by 74%. In addition to that decreasing the length of the dust shield in the presence of the air nozzle has decreased the deposition rate by 88%. Therefore, it can be concluded that the air nozzle has a significant effect on deposition rate of particles, and this effect can be enhanced by decreasing the dust shield length.

**Table 4:** Influence of different mitigation techniques on the dust deposition rate for a dust particle size of 90 μm

	Deposition rate*, *λ*	%Decrease in deposition rate, *%Δλ* = (*λ*_*s*_*-λ/λ*_*s*_) × 100
No shield (**λ**_**s**_)	1.53%	0%
Dust shield of length L	0.86%	44%
Dust shield of length L + extension plate + no mm air gap	0.75%	50%
Dust shield of length L + extension plate + 60 mm air gap	0.39%	74%
Dust shield of length 2/3L + extension plate + 60 mm air gap	0.18%	88%

*λ_*s*_ is the dust deposition rate in case of no shield.

The loss in the panel output due to dust deposition is proportional to the dust deposition density, which is dependent on the dust deposition rate, the exposure time of the panel to dust and area of the panel (Jiang et al. 2011; Lu et al. 2016). Therefore, it is quite difficult to calculate loss in the panel output, since the present study is a 2D simulation, where there is no definition for a surface area of the panel and the exposure time. Accordingly, the dust deposition density cannot be estimated. Therefore, further research should be done to extend the 2D simulations to 3D, in order to infer the power loss due to dust deposition. Another important parameter that should be taken into consideration is the side winds which have a great influence on the dust deposition rate (Eisa et al. 2023). It has been found that the side winds assist in removing or blowing off landed dust particles on the panel’s surface. Therefore, it is recommended to perform 3D simulations in order to study the effect of side winds on the dust deposition rate on the panel’s surface. It can be concluded from the presented research that the use of a dust shield will affect the output of the panel, however, further research should be conducted to determine experimentally the influence of the dust shields and air nozzles on the dust deposition rate and to validate experimentally the presented numerical results.

## Conclusions and recommendations for future work

The objective of this study was to minimize dust accumulation on PV panels operating street light posts using dust shields. Numerical simulations have been performed in order to infer the influence of using different designs of dust shields on the dust deposition rate on the panel’s surface. The airflow around a PV panel as well as the dust deposition rate were numerically investigated as a function of the applied dust shield. The following conclusions can be drawn based on the present study:

1. A dust shield having the same dimensions of the PV panel and subtending at an angle of 120° with the panel decreases the dust deposition rate by more than 44%.
2. Adding an extension plate to the PV panel in the presence of a dust shield, such that an air gap exists between the shield and the added plate, acts as an air nozzle that decreases the deposition rate by more than 84%.
3. The combined effect of using a dust shield less than the panel’s length by one third and a nozzle of a gap thickness of 60 mm decreases the dust deposition rate by more than 88%.

However, this new developed dust mitigation technique needs to be further studied, such that the following points are recommended for future work:

1. Performing 3D numerical simulations in order to study the power loss due to dust deposition in addition to the influence of the side winds.
2. Studying experimentally the effect of the air gap size on the dust accumulation rate.
3. Further research should be performed to find out the optimum air gap thickness that minimizes the dust deposition rate on the panel’s surface and maximizes cooling of the panel.

## Data Availability

The authors confirm that all data related to this research is presented in the submitted paper, and any further data will be made available upon request.

## Abbreviations

CFD: Computational fluid dynamics
DPM: Discrete phase model
DRW: Discrete random walk
FVM: Finite volume method
MENA: Middle East and North Africa
PV: Photovoltaic
RANS: Reynolds averaged Navier–Stokes
Re: Reynolds number
SST: Shear stress transport
TKE: Turbulent kinetic energy

## Nomenclature

*A*_*p*_: Particle cross-sectional area
*C*_*D*_: Drag coefficient
*C*_*p*_: Mean pressure coefficient
*D*_*ω*_: Cross-diffusion term
$\overrightarrow{F}$: Saffman’s lift force
*G*_*k*_: Generation of turbulent kinetic energy *k*
*G*_*ω*_: Generation of ω
*g*: Acceleration of gravity
*H*_*P*_: Height of the panel
*L*: Length
*m*_*p*_: Particle mass
*N*_*d*_: Number of deposited dust particles
*N*_*P*_: Number of injected dust particles
$\overline{p }$: Time-averaged pressure
*S*_*k*_: User-defined source terms of *k*
*S*_*ω*_: User-defined source terms of ω
${u}_{i}$: Time-averaged velocity
*u*_*p*_: Particle velocity
*V*: Air velocity
*V*_*p*_: Particle volume
*W*: Distance measured along the solar panel width from the panel’s leading edge
*W*_*PV*_: Solar panel width
*Y*_*k*_: Dissipation rates of *k*
*Y*_*ω*_: Dissipation rates of *ω*

## Greek symbols

*Γ*_*k*_: Effective diffusivity of *k*
*Γ*_*ω*_: Effective diffusivity of *ω*
*λ*: Dust deposition rate
*λ*_*s*_: Dust deposition rate in case of no shield
*μ*: Dynamic viscosity
$v$: Fluid kinematic viscosity
*ρ*: Density
*ρ*_*p*_: Particle density
$\rho {u}_{i}^{^{\prime}}{u}_{j}^{^{\prime}}$: Reynolds stress tensor
